# Case Report: Two-patch technique with BioGlue salvages a patient with acute inferior wall myocardial infarction complicated by ventricular septal rupture and cardiogenic shock

**DOI:** 10.3389/fcvm.2025.1666868

**Published:** 2025-10-07

**Authors:** Rei-Yeuh Chang, Tsung-Hsien Chen, Han-Lin Tsai, Yin-Chia Chen

**Affiliations:** ^1^ Division of Cardiology, Department of Internal Medicine, Ditmanson Medical Foundation Chia-Yi Christian Hospital, Chiayi, Taiwan; ^2^ Department of Internal Medicine, Ditmanson Medical Foundation Chia-Yi Christian Hospital, Chiayi, Taiwan; ^3^ Department of Cardiovascular Surgery, Ditmanson Medical Foundation Chia-Yi Christian Hospital, Chiayi, Taiwan

**Keywords:** acute myocardial infarction, ventricular septal rupture, cardiogenic shock, post-infarction ventricular septal rupture, cardiac surgery

## Abstract

Post-myocardial infarction ventricular septal rupture (VSR) is a rare but often fatal complication of acute myocardial infarction (AMI). Without surgical or percutaneous intervention, mortality is exceedingly high. Even with corrective procedures such as surgical repair or transcatheter septal closure, in-hospital mortality remains substantial, particularly in hemodynamically unstable patients. We report a case of acute inferior–posterior wall ST-segment elevation myocardial infarction complicated by a large VSR and cardiogenic shock. Immediate venoarterial extracorporeal membrane oxygenation support was initiated. The patient subsequently underwent surgical repair using a modified infarct exclusion technique, in which BioGlue was applied between two patches to reinforce closure, and the second patch was extended into the ventriculotomy to simplify the procedure. The patient survived and remained free of recurrent VSR at the 5-month follow-up. This modified approach offers a feasible and effective strategy for managing acute inferior–posterior VSR following AMI, particularly in critically ill patients.

## 1 Introduction

Post-infarction ventricular septal rupture (PIVSR) is one of the most devastating mechanical complications of acute myocardial infarction (AMI). The ventricular septum is primarily supplied by septal perforating branches, and occlusion of either the left anterior descending artery or the posterior descending artery may result in PIVSR. The incidence has decreased from 1% to 3% in the pre-reperfusion era to 0.17%–0.21% following the widespread use of early thrombolysis and primary percutaneous coronary intervention (PCI) (1, 2). PIVSR creates a left-to-right shunt, leading to pulmonary congestion, progressive heart failure, and cardiogenic shock. Conservative medical management is almost universally fatal; in the GUSTO-I trial, the 30-day mortality reached 94% with medical therapy alone (3).

Surgical repair remains the standard of care and is potentially life-saving, but perioperative mortality remains high. According to the Society of Thoracic Surgeons database, in-hospital or 30-day mortality after PIVSR repair is approximately 43% (4). Even after successful repair, recurrent septal defects and bleeding complications may occur. Transcatheter septal closure has emerged in recent years as an alternative therapeutic approach. Systematic reviews report in-hospital mortality rates of 27%–32% (5, 6), although this strategy is also limited by complications such as arrhythmia, ventricular perforation, and residual shunting. Overall, both surgical and percutaneous strategies have shown limited improvement in outcomes.

We present a case of acute inferior wall ST-segment elevation myocardial infarction (STEMI) complicated by a large PIVSR and cardiogenic shock. The patient was stabilized with venoarterial extracorporeal membrane oxygenation (VA-ECMO) and underwent emergent surgical repair using a modified infarct exclusion technique reinforced with biologic glue. This approach successfully achieved durable closure of the large defect without residual shunt.

## 2 Case description

A 71-year-old man with a history of lumbar neuralgia under regular follow-up at a local clinic presented with intermittent, non-radiating chest pain for 1 week, located at the mid-sternum (pain score 3–4/10). On 24 March 2025, he developed chest pain associated with diaphoresis and weakness, without specific aggravating or relieving factors. He was transferred to our emergency room (ER) due to worsening symptoms.

**Figure 1:**
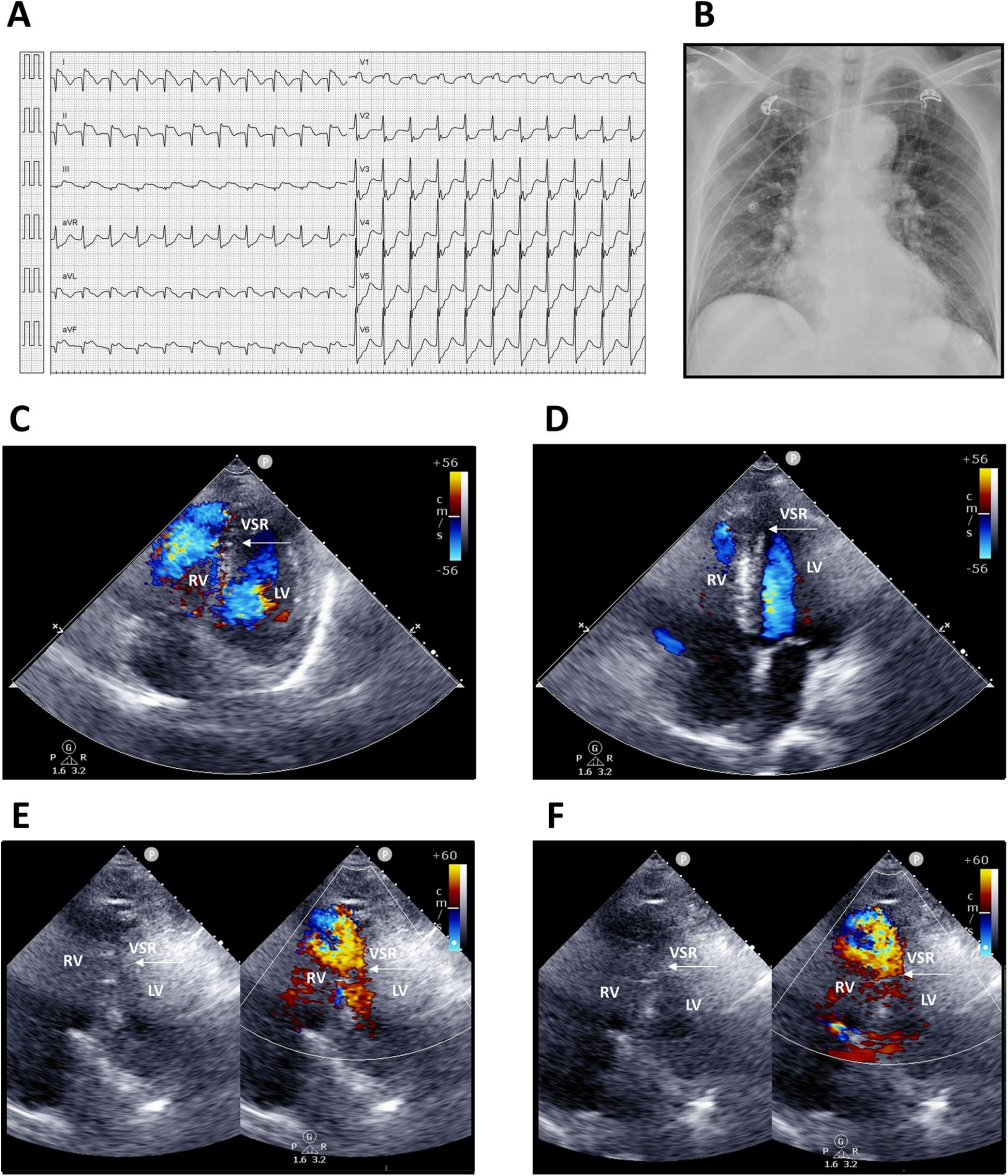
Electrocardiography, chest radiography, and transthoracic echocardiography findings. **(A)** Twelve-lead electrocardiogram showing supraventricular tachycardia 130 bpm; incomplete right bundle branch block (ICRBBB); ST-segment elevation in leads I, II, and aVF (suspected reversed arm leads); and ST-segment depression in V2–6. **(B)** Chest radiograph demonstrating right ventricular enlargement and pulmonary arterial plethora. **(C**,**D)** Apical four-chamber views. **(E**,**F)** Modified apical four-chamber views with two-dimensional and color Doppler imaging, revealing a large rupture of the inferior–posterior interventricular septum (arrows) with a significant left-to-right shunt. LV, left ventricle; RV, right ventricle; VSR, ventricular septal rupture.

Upon arrival, he was conscious but in shock, with vital signs showing blood pressure 68/48 mmHg, heart rate 136 bpm, respiratory rate 20/min, and body temperature 35.4°C. Physical examination revealed a grade 3/6 pansystolic murmur along the left sternal border, with clear breath sounds. Laboratory findings demonstrated leukocytosis [white blood count (WBC) 12,150/µL, segmented neutrophils 95.8%] and elevated troponin-I (9.873 ng/mL). Electrocardiography showed supraventricular tachycardia, complete right bundle branch block (CRBBB); ST-segment elevation in leads I, II, and aVF (reversed arm leads); and ST-segment depression in V2–6 (Figure 1A). An acute inferior wall ST-elevation myocardial infarction was suspected. Chest radiography revealed right ventricular enlargement and pulmonary plethora (Figure 1B). Based on these findings, an acute inferior STEMI was diagnosed. Aspirin, ticagrelor, and intravenous heparin were administered.

**Figure 2:**
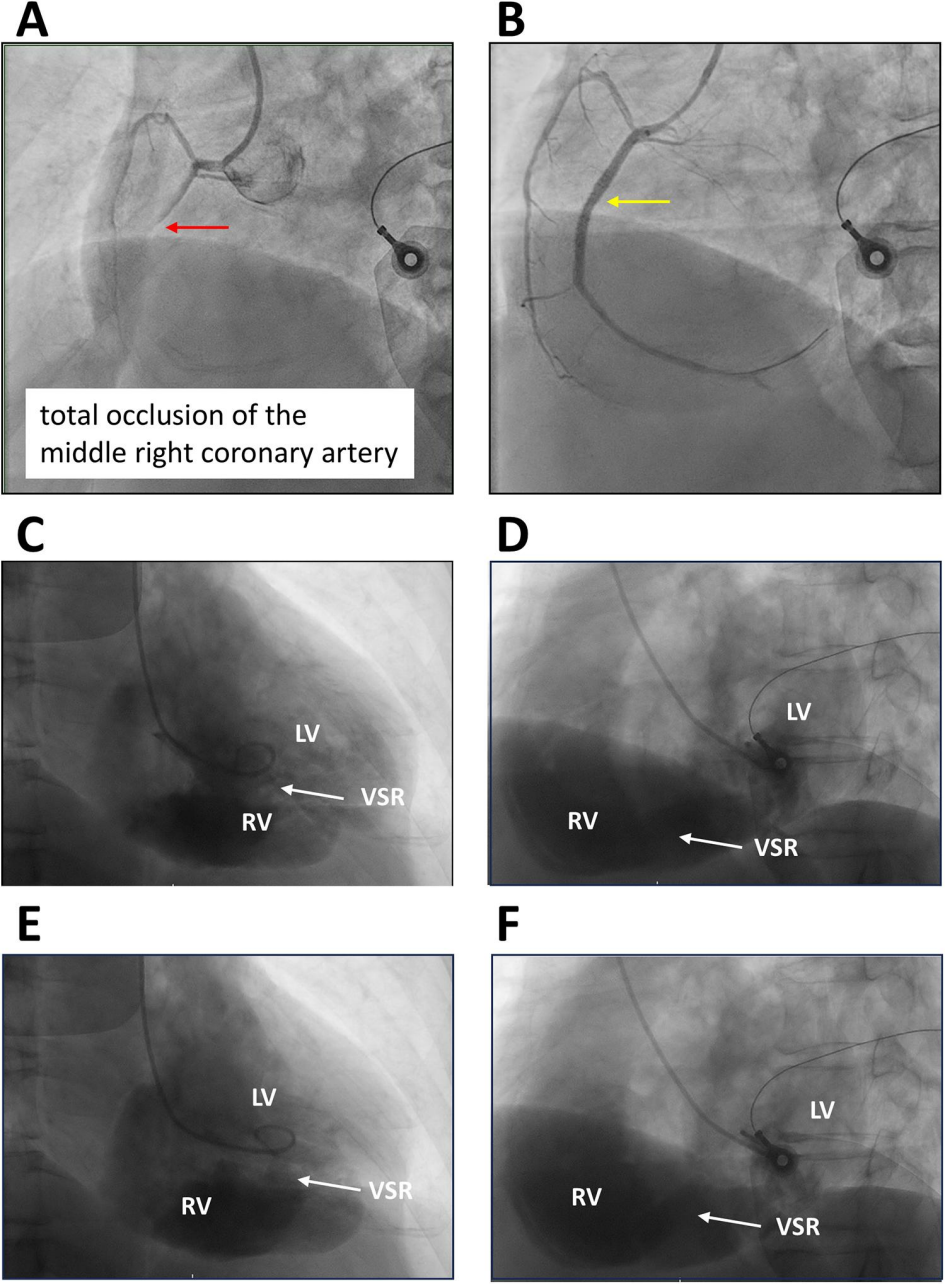
Coronary and left ventricular angiography. **(A**,**B)** Left anterior oblique (LAO) view with cranial angulation demonstrating total occlusion of the middle right coronary artery (red arrow) and subsequent bare-metal stent deployment (yellow arrow). **(C**,**D)** Right anterior oblique (RAO) and LAO views during diastole. **(E**,**F)** RAO and LAO views during systole, illustrating rupture of the inferior and posterior interventricular septum with a substantial left-to-right ventricular shunt (arrows). LV, left ventricle; RV, right ventricle; VSR, ventricular septal rupture.

Transthoracic echocardiography suggested distal interventricular septum rupture with a left-to-right shunt (Figures 1C–F). Intravenous normal saline was given for hypotension, and the patient underwent emergency coronary angiography, which revealed total occlusion of the middle right coronary artery. Balloon angioplasty with bare-metal stenting was performed (Figures 2A,B; Supplementary Videos S1–S6). Left ventriculography confirmed a large rupture in the posterior inferior interventricular septum with significant left-to-right shunting (Figures 2C–F; Supplementary Videos S7, S8). VA-ECMO was initiated for hemodynamic stabilization, after which he was admitted to the intensive care unit (ICU). Informed consent for surgical intervention was obtained from his family.

**Figure 3:**
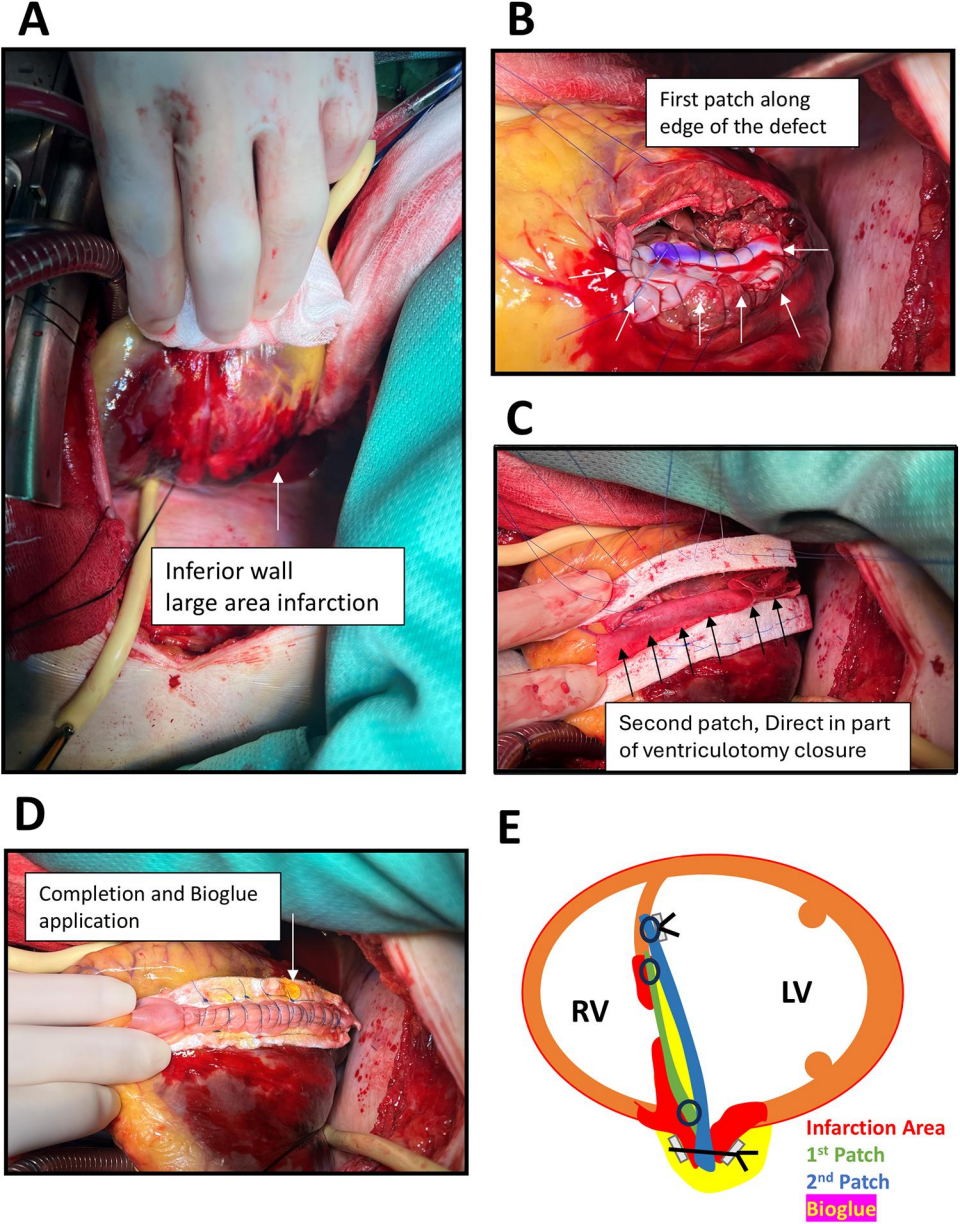
Operative findings and procedures. **(A)** Medium sternotomy with posterior left ventriculotomy along the septal margin, revealing extensive infarction of the inferior wall (arrow) and a large tear of the posterior–inferior basal septum measuring 1.5 cm × 6 cm. **(B)** Placement of the first bovine pericardial patch using continuous 4-0 Prolene suture (arrows). **(C)** Application of a second patch composed of two layers of bovine pericardium combined with a 32 mm J graft, employing the infarct exclusion technique, with BioGlue applied between the two layers (arrows). **(D)**) Closure of the ventriculotomy with additional applied BioGlue reinforcement (arrow). **(E)** Schematic illustration of the novel operative technique.

Approximately 4 h after ER presentation, the patient underwent surgery. Intraoperative transesophageal echocardiography confirmed the location and extent of the rupture. Median sternotomy and posterior left ventriculotomy were performed 1.5 cm from the septal margin. A 1.5 cm × 6 cm longitudinal tear in the posterior inferior basal septum with extensive inferior wall infarction was identified (Figure 3A). Repair was achieved using a modified infarct exclusion technique. The first bovine pericardial patch was sutured with running 4-0 Prolene along the tear margin. A second two-layer patch (bovine pericardium + J graft, 32 mm straight graft cut to form a patch) was anchored with interrupted pledgeted 3-0 Prolene over a relatively healthy septum, leaving the ventriculotomy edge unsutured. BioGlue was applied between the two patches using a gauze barrier to prevent embolization (Figures 3B,C). The second patch extended 2 cm beyond the ventriculotomy edge, which was then closed with two double-layer felt-reinforced 3-0 Prolene sutures. Additional BioGlue was applied for reinforcement (Figures 3D,E). Post-repair transesophageal echocardiography confirmed no residual shunt. The patient was weaned from cardiopulmonary bypass to VA-ECMO, and an intra-aortic balloon pump (IABP) was inserted via the left femoral artery.

On postoperative day 2, the patient developed hypovolemic shock due to cardiac tamponade. Exploratory sternotomy revealed diffuse bone marrow oozing and approximately 1,500 mL mediastinal hematoma. Hemostasis was achieved with Surgicel packing. Subsequent echocardiography demonstrated preserved cardiac output, a non-dilated right ventricle, and intact VSR repair. His condition gradually improved, VA-ECMO was removed on postoperative day 3, and he was discharged on day 20.

At discharge, medications included clopidogrel 75 mg daily and warfarin 0.5 mg daily [for thromboembolism prevention related to the left ventricular (LV) patch], spironolactone 25 mg daily, intravenous furosemide 20 mg every 12 h, bisoprolol 5 mg daily (for heart failure), and amiodarone 200 mg daily (for paroxysmal atrial fibrillation). Follow-up echocardiography at 1 month showed no residual VSR. At 5 months postoperatively, the patient remained alive and in good recovery.

## 3 Discussion

PIVSR is a rare but highly lethal complication of myocardial infarction. Surgical repair or transcatheter septal closure are the only definitive treatment options, yet mortality remains high, particularly in hemodynamically unstable patients (1, 2). Survival without surgical intervention is <10%. Under medical therapy alone, approximately 25% of patients with cardiogenic shock die within 24 h, and >50% die within 1 week of rupture (7). Notably, 50%–60% of patients present in cardiogenic shock, necessitating immediate surgical intervention. Only approximately 5% are hemodynamically stable enough to allow delayed surgery until fibrotic tissue formation, while those in an intermediate condition are typically recommended to undergo surgery within 12–24 h (7, 8). However, emergent surgery carries substantial risk, including myocardial bleeding and residual shunting.

Guideline recommendations differ regarding the timing of surgery. The European Society of Cardiology advocates delaying repair in hemodynamically stable patients when supported by medical therapy or mechanical circulatory support (MCS), allowing time for septal fibrosis. In contrast, the American College of Cardiology suggests deferring corrective treatment beyond 7–10 days in stable patients (9). MCS may serve as a bridge to surgery in patients with shock. IABP reduces LV afterload and augments coronary perfusion; however, the IABP-SHOCK trial demonstrated no survival benefit in AMI-related cardiogenic shock (10). VA-ECMO provides systemic circulatory support, enhances tissue oxygenation, and unloads the distended right ventricle, thereby stabilizing patients prior to surgery. Percutaneous microaxial transaortic assist devices (e.g., Impella) have been shown to reduce ventricular shunting and pulmonary congestion (11), but isolated left ventricular assist devices (LVADs) are less effective in PIVSR due to inadequate LV volume caused by left-to-right shunting (12). Combined LVAD and VA-ECMO support may be considered in cases with concomitant LV dysfunction, although this was not necessary in our patient. In the present case, VA-ECMO provided effective hemodynamic stabilization without complications.

Another therapeutic option for PIVSR is transcatheter closure. Initially, this technique was reserved as a palliative measure for patients deemed unsuitable for surgery. With growing expertise, its use has expanded to selected patients with favorable septal anatomy. However, certain morphologies—such as posterior PIVSR in close proximity to the mitral valve, apical defects, complex serpiginous tracts, and defects adjacent to valvular structures—remain less suitable for percutaneous closure (9). In a meta-analysis, residual shunting necessitated surgical repair in 16.1% of patients undergoing transcatheter closure, while 7.8% of surgical patients required subsequent transcatheter intervention (13). Reported in-hospital mortality of transcatheter PIVSR closure ranges from 32% to 55% (6, 13). Ultimately, the choice between surgical and transcatheter approaches depends on local expertise, patient hemodynamic stability, and the anatomical characteristics of the defect.

The most commonly employed surgical techniques for PIVSR include the infarct excision technique (Daggett), the infarct exclusion technique (David), and the apical amputation technique (Daggett). Daggett initially described infarct excision, which involves infarctectomy and reconstruction of the interventricular septum and infarcted ventricular wall using Dacron patches. Daggett also proposed apical amputation for apical ruptures. In contrast, David introduced the infarct exclusion technique, which avoids infarctectomy by suturing a pericardial patch to the endocardium of the non-infarcted left ventricle.

Comparative studies suggest superior outcomes with the exclusion technique. Lundblad and Abdelnoor (14) demonstrated that David's approach resulted in improved early and late survival compared with Daggett's direct septal closure. The exclusion method is particularly advantageous in patients with extensive infarction, especially posterior ruptures. Reported operative mortality is approximately 10% in hemodynamically stable patients but significantly higher in those with cardiogenic shock (15). In one series, the infarct exclusion technique was associated with a hospital mortality rate of 31.2% (8). Matteucci et al.'s (16) meta-analysis reported an overall operative mortality of 38.2% across two eras (1971–2000 vs. 2001–2018), with predictors of poor outcome including preoperative or perioperative IABP support, right ventricular dysfunction at presentation, posterior defects, and emergent repair.

Despite its advantages, David's method remains limited by risks of residual shunting, uncontrolled bleeding, and recurrent rupture. To address these challenges, Iino et al. (17) developed a two-patch technique reinforced with BioGlue for anterior PIVSR repair. In this method, the first bovine pericardial patch is sutured to the endocardium surrounding the defect, and a second, larger patch is anchored further out, with BioGlue applied along the suture line between the two patches. In their series of seven patients, the technique achieved 0% in-hospital mortality with no residual shunt.

Repair of posterior or inferior septal ruptures is more challenging due to restricted anatomical access. We adopted a modification of Iino et al.'s two-patch method for a case of inferior PIVSR following AMI. BioGlue was applied between the two layers, within the cavity between them, and along the closed ventriculotomy surface to reinforce suture lines and reduce the risk of blood leakage. This strategy provided a strong adhesive layer that may prevent late recurrence caused by suture site tearing. While BioGlue carries a theoretical risk of embolization, application between two patches avoided direct exposure to circulating blood, thereby minimizing this risk.

Another modification simplified the exclusion method by extending the edge of the second patch into the ventriculotomy closure, effectively “sandwiching” it within the suture line. This approach ensured a secure repair and was technically easier than folding the patch into the opposite endocardial surface of the ventriculotomy. Postoperative echocardiography and follow-up have confirmed a durable repair without residual shunting.

The patient presented with cardiogenic shock and supraventricular tachycardia. Following cardiac catheterization, VA-ECMO support was initiated, and surgical repair was performed the same day. Given the patient's unstable hemodynamics and the presence of a large posterior–inferior defect unsuitable for transcatheter closure, early surgical intervention was mandatory. Although early surgery carries inherent risks of bleeding and residual shunting, these complications were successfully avoided using a modified infarct exclusion technique with a double-patch repair reinforced by BioGlue.

## 4 Limitations

This report has several limitations. First, PIVSR is rare, and thus the surgical experience is limited to a single surgeon at a single institution. No direct comparison with alternative surgical techniques was possible, and our institutional experience with transcatheter closure is also limited. Larger case series are needed to allow meaningful comparisons between surgical and percutaneous approaches. Second, in patients with PIVSR complicated by cardiogenic shock, we were able to provide IABP and VA-ECMO support prior to surgery; however, microaxial transaortic assist devices such as Impella are not reimbursed by the national health insurance system in our country, which limits their applicability due to high cost. Third, the present patient has been followed for only 5 months and remains alive without recurrence, but longer follow-up is necessary to confirm the durability of the repair.

## 5 Conclusion

In a patient with an acute inferior myocardial infarction complicated by large inferior posterior VSR and cardiogenic shock, MCS with VA-ECMO and, where available, microaxial transaortic devices can provide critical stabilization prior to surgery. Definitive repair using a modified two-patch infarct exclusion technique reinforced with BioGlue may achieve successful closure without residual shunting. Additional clinical experience and longer follow-up are needed to validate the efficacy and durability of this approach.

## Data Availability

The original contributions presented in the study are included in the article/Supplementary Material, further inquiries can be directed to the corresponding author.

## Supplementary material

Supplementary Videos S1, S2 Left coronary angiography (RAO caudal and LAO cranial view) demonstrating 70% stenosis of the middle left anterior descending artery and 90% stenosis of the distal left circumflex artery.

Supplementary Videos S3, S4 Right coronary angiography (RAO and LAO views) showing total occlusion of the middle right coronary artery.

Supplementary Videos S5, S6 Post-intervention right coronary angiography (LAO views) after balloon angioplasty and bare-metal stent placement, demonstrating TIMI grade 3 flow.

Supplementary Videos S7, S8 Left ventricular angiography (RAO and LAO views) revealing rupture of the inferior and posterior interventricular septum with a large left-to-right ventricular shunt.
